# Economic Decisions with Ambiguous Outcome Magnitudes Vary with Low and High Stakes but Not Trait Anxiety or Depression

**DOI:** 10.5334/cpsy.79

**Published:** 2021-10-21

**Authors:** Tomislav D. Zbozinek, Caroline J. Charpentier, Song Qi, Dean Mobbs

**Affiliations:** 1 Division of Humanities and Social Sciences, California Institute of Technology, 1200 E. California Blvd., MC 228-77, Pasadena, CA 91125, US; 2 National Institute of Mental Health, 6001 Executive Boulevard, Room 6200, MSC 9663, Bethesda, MD 20892, US; 3 California Institute of Technology, Humanities and Social Sciences, 1200 E. California Blvd., MC 228-77, Pasadena, CA 91125, US

**Keywords:** anxiety, depression, behavioral economics, ambiguity, prospect theory

## Abstract

Most of life’s decisions involve risk and uncertainty regarding whether reward or loss will follow. Decision makers often face uncertainty not only about the likelihood of outcomes (what are the chances that I will get a raise if I ask my supervisor? What are the chances that my supervisor will be upset with me for asking?) but also the magnitude of outcomes (if I do get a raise, how large will it be? If my supervisor gets upset, how bad will the consequences be for me?). Only a few studies have investigated economic decision making with ambiguous likelihoods, and even fewer have investigated ambiguous outcome magnitudes. In the present report, we investigated the effects of ambiguous outcome magnitude, risk, and gains/losses in an economic decision-making task with low stakes (Study 1; $3.60–$5.70; N = 367) and high stakes (Study 2; $6–$48; N = 210) using a within-subjects design. We conducted computational modeling to determine individuals’ preferences/aversions for ambiguous outcome magnitudes, risk, and gains/losses. We additionally investigated the association between trait anxiety and trait depression and decision-making parameters. Our results show that increasing stakes increased ambiguous gain aversion and unambiguous risk aversion but increased ambiguous sure loss preference; participants also became more averse to ambiguous sure gains relative to unambiguous risky gains. There were no significant effects of trait anxiety or trait depression on economic decision making. Our results suggest that as stakes increase, people tend to avoid uncertainty in the gain domain (especially ambiguous gains) but prefer ambiguous vs unambiguous sure losses.

Prospect theory is a well-supported economic model of decision making under risk, offering a promising avenue through which we can understand the role of uncertainty in decision making (Abdellaoui et al., 2008, 2016; Kahneman et al., 1991; Kahneman & Tversky, 2013; Tversky & Kahneman, 1992). Prospect theory suggests that decision making can be explained by risk aversion (i.e., the tendency to prefer certain options over uncertain options) and loss aversion (i.e., the tendency to weigh potential losses more strongly than potential gains, usually at a 2:1 ratio). Importantly, most economic decision-making studies use known gain/loss values and manipulate the known likelihood of receiving each (i.e., the likelihood and magnitude of gains/losses are unambiguous). However, conducting studies in which the likelihood or magnitude of gains/losses are ambiguous increases ecological validity since most real-world decisions involve both ambiguous likelihood and magnitude. Using an economic example, when making an investment in the stock market, the likelihood that gains or losses will occur is ambiguous, as is the magnitude of those potential gains and losses. Similarly, using a non-economic example, for an individual deciding whether to ask someone on a date, the magnitude of negative outcomes (e.g., politely getting declined or getting harshly rejected) or positive outcomes (e.g., going on one date, several dates, or entering a long-term relationship) and the likelihood of each occurring are ambiguous. Thus, incorporating ambiguity into economic decision-making studies may prove to be insightful and more ecologically relevant.

A small subset of economic decision-making studies manipulate ambiguity regarding the likelihood that the individual will receive a gain or loss of known magnitude, and both theoretical (Camerer & Weber, 1992) and empirical (Feldman-Hall et al., 2016; Fox & Tversky, 1995; Heath & Tversky, 1991; Huettel et al., 2006; Ruderman et al., 2016) work suggests that individuals show aversion to ambiguous outcome likelihoods. One hypothesis (Fox & Tversky, 1995) as to why this occurs is that individuals prefer relative competence to relative ignorance – meaning, when faced with an option of ambiguous likelihood vs an option of unambiguous likelihood, individuals usually prefer the unambiguous option because of certainty regarding the likelihood of that outcome. While it is more common for studies to manipulate ambiguous outcome likelihood (Camerer & Weber, 1992), a few studies have manipulated ambiguity regarding the magnitude of an outcome (i.e., uncertainty regarding how small/large the gain/loss will be). Overall, previous experiments tend to show aversion to ambiguous gains (González-Vallejo et al., 1996; Kuhn & Budescu, 1996; Oliver, 1972) but potential preference for ambiguous losses (Ho et al., 2002). However, many of these experiments operationalize the ambiguous outcome as an unambiguous range of values from which the true outcome could be drawn (e.g., outcome is between $10 to $20 vs outcome is exactly $15). An approach that would presumably increase ambiguity and provide more ecological validity would be to provide no explicit information regarding the value of the ambiguous outcome on a given trial, much like no explicit information is given regarding the potential outcomes of asking someone on a date. Thus, a major goal of the present report is to investigate the effects of ambiguous/unambiguous outcome magnitudes with both risk/no risk and gains/losses; as a novel feature of our experiment, we assess ambiguous gains and ambiguous losses without explicitly stating the range of possible values on a given choice but rather just inform the participant whether the ambiguous value is a gain or a loss. ***Figure 1*** depicts our modified prospect theory model with outcome ambiguity, in which ambiguity aversion or preference is depicted by a multiplicative weight – much like the loss aversion parameter from traditional prospect theory (see our repository at *https://github.com/tzbozinek/economic-decision-making-ambiguity* for an interactive figure of our model titled “Zbozinek et al – Prospect Theory Ambiguity Model.html”).

**Figure 1:**
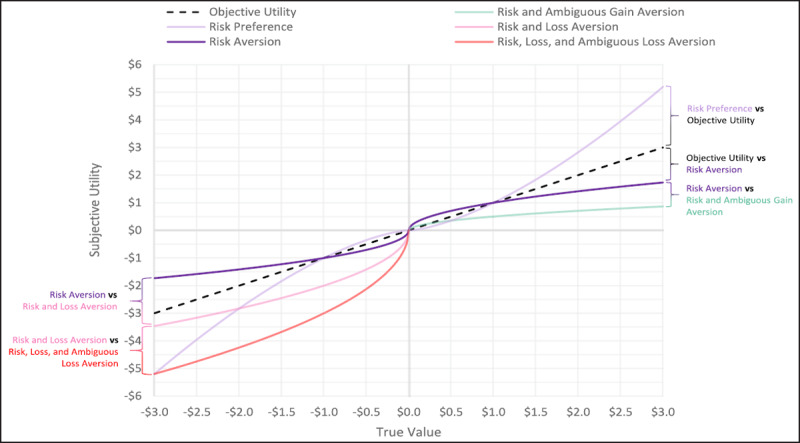
Ambiguous Outcome Magnitude Prospect Theory Model. Prospect theory includes (unambiguous) risk aversion/preference (ρ) and (unambiguous) loss aversion/preference (λ). Our model builds upon prospect theory by additionally parameterizing preference/aversion towards ambiguous outcome magnitudes (α). In this figure, we separately parameterize ambiguous gain aversion (αG) and ambiguous loss aversion (αl) for concision (akin to Model 3; see Methods). For illustrative purposes, we show Objective Utility (depicting rational decision making), risk preference, risk aversion, loss aversion, aversion of ambiguous loss magnitudes, and aversion of ambiguous gain magnitudes. We show additive effects of parameters (e.g., risk aversion vs risk aversion and ambiguous gain aversion). The model shows that risk preference/aversion affects the curvature of the subjective utility function (i.e., via its exponential calculation); (unambiguous) loss aversion, ambiguous loss aversion, and ambiguous gain aversion shift the curve up/down (i.e., via their multiplicative calculation; see Table 2). For unambiguous “True Values,” the monetary value of the gamble is explicitly known; for ambiguous “True Values,” the monetary value represents the most likely or central value of the ambiguous values (e.g., mean). Parameter values were 1 unless otherwise specified here: Risk Preference (ρ = 1.5); Risk Aversion (ρ = .5); Risk and Ambiguous Gain Aversion (ρ = .5, αG = .5); Risk and Loss Aversion (ρ = .5, λ = 2); and Risk, Loss, and Ambiguous Loss Aversion (ρ = .5, λ = 2, αL = 1.5). Please see our repository for an interactive figure of this model, where the user can change inputs (e.g., risk preference) and observe the outputs (file is titled “Zbozinek et al – Prospect Theory Ambiguity Model.html”): *https://github.com/tzbozinek/economic-decision-making-ambiguity*.

Moreover, an interesting question is whether ambiguous outcome preference/aversion is stable or varies depending on monetary stakes. Using the dating example, perhaps asking someone on a date who is very emotionally expressive would be akin to a “high stakes” decision (where the acceptance or rejection could be very emphatic), whereas asking someone on a date who is less emotionally expressive is akin to a “low stakes” decision (where the acceptance or rejection could be less emphatic). Importantly, people’s decision-making in low and high stakes situations might differ, and their preference/aversion for risk, loss, and ambiguity may change. Previous studies have shown that risk aversion increases with greater monetary stakes (Binswanger, 1980; Fehr-Duda et al., 2010; Harrison et al., 2005; Holt & Laury, 2002, 2005; Kachelmeier & Shehata, 1992; Markowitz, 1952), which could be due to a decrease in perceived probability of receiving gains as gain value increases (Fehr-Duda et al., 2010). Because risk and ambiguity are both forms of uncertainty, we suspect that ambiguity aversion would likely increase with greater monetary stakes much like risk aversion, though this may be reversed for losses (Ho et al., 2002). Along the same reasoning (Fehr-Duda et al., 2010), perhaps as unambiguous gain value increases, perceptions may relatively decrease regarding the amount the individual will receive from choosing an ambiguous gain option. To test this, we conducted two experiments of the same economic decision-making task but varied the amount of money that could be gained/lost on a given decision by a factor of 20 (see Methods for details). Additionally, participants’ decisions actually affected their payment (rather than making hypothetical decisions), greatly adding to the validity of our design (Fehr-Duda et al., 2010; Holt & Laury, 2002). To our knowledge, this is the first report to assess in a within-subjects design how risk/no-risk, gains/losses, and unambiguous/ambiguous outcome magnitudes affect decisions, as well as the effect of incentive-compatible low vs high stakes (i.e., not hypothetical).

Furthermore, there may be individual differences in economic decision-making related to emotional disposition. Anxiety and depression have shown mixed associations with risk and loss aversion (Baek et al., 2017; Chandrasekhar Pammi et al., 2015; Chapman et al., 2007; Charpentier et al., 2017; Ernst et al., 2004; Harpaz-Rotem et al., 2017; Lauriola & Levin, 2001; Leahy et al., 2012; Lerner & Keltner, 2000, 2001; Maner et al., 2007; Raghunathan & Pham, 1999; Sip et al., 2018; Smoski et al., 2008), but we suspect that anxious or depressed individuals may show ambiguity aversion akin to “catastrophizing” in clinically anxious and depressed individuals (i.e., overestimating the magnitude of future negative outcomes or underestimating the magnitude of future positive outcomes) (Beck et al., 1979; Beck, 1967; Beck et al., 1974). Anxiety is consistently associated with increased negative affect (Brown et al., 1998; Prenoveau et al., 2010; Watson, 2005) and avoidance of objectively safe situations for fear of negative outcomes (Craske et al., 2012; Gazendam et al., 2013). Due to anxiety’s association with negative affect and fear of negative outcomes, perhaps anxious individuals have an aversion to ambiguous losses. Conversely, depression is consistently associated with increased negative affect and decreased positive affect (Brown et al., 1998; Prenoveau et al., 2010; Watson, 2005), reduced anticipation of positive outcomes (Berlin et al., 1998; Clepce et al., 2010; Holmes et al., 2009; Keedwell et al., 2005; MacLeod & Salaminiou, 2001; Treadway et al., 2012; Vrieze et al., 2013; Wacker et al., 2009) and loss (Leahy, 2002; Leahy, 1997). Due to depression’s association with low positive affect and low anticipation of positive outcomes, perhaps depressed individuals may have an aversion of ambiguous gains; similarly, depression’s association with high negative affect and loss suggest it may be associated with aversion of ambiguous losses. Our report is the first to assess the association between anxiety, depression, and ambiguous outcome magnitudes in a controlled economic decision-making experiment.

Overall, the present report addresses four main aims: 1) determine the effect of ambiguous outcome magnitude on decision making, 2) investigate whether ambiguous outcome magnitude is a separable construct from risk and loss (assessed by modeling ambiguity preference/aversion separately from risk preference and loss aversion to investigate whether it improves model fit), 3) investigate the effect of low vs high stakes on decision making with risk/no-risk, gains/losses, and ambiguity/no-ambiguity, 4) assess the association between trait anxiety and trait depression and economic decision making. We investigate these aims below in our pre-registered experiments (Study 1: *https://osf.io/2k68e*; Study 2: *https://osf.io/vzypm*).

## Results

**Figure 2:**
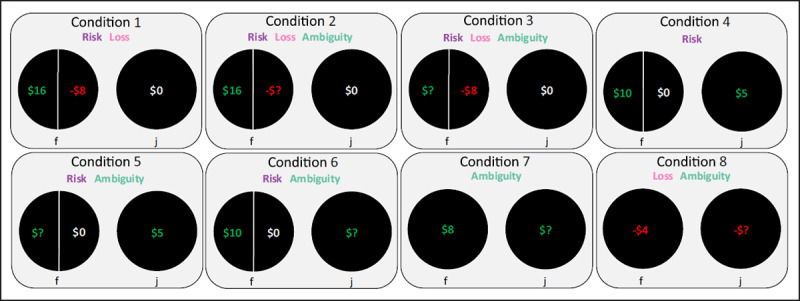
Experimental Conditions. This figure presents a schematic of each condition. Gains are color-coded with green text, losses with red text, and $0 with white text. Green question marks (i.e., “$?”) indicate ambiguous gain magnitude; red question marks (i.e., “–$?”) indicate ambiguous loss magnitude. Starting payments were $4.50 (Study 1) or $24 (Study 2), with total possible payments ranging $3.60 to $5.70 (Study 1) and $6 to $48 (Study 2). Unambiguous gains ranged from $.05 to $1.20 (Study 1) or $1 to $24 (Study 2). Unambiguous losses ranged from –$.05 to –$.90 (Study 1) or –$1 to –$18 (Study 2). Circles without a vertical line indicate a 100% chance of receiving that outcome. Circles with a vertical line indicate a 50%/50% chance of receiving each outcome. Conditions that involve Risk, Loss, or Ambiguity are indicated within each condition’s box.

Our experiment included eight conditions. Conditions 1 and 4 contained exclusively unambiguous outcomes, whereas the remaining six conditions contained a choice with an ambiguous outcome. Participants were presented with two options on a given trial and had 5 seconds to make a binary choice to select either the left or right option. Upon making the decision, participants continued to the next trial pseudo-randomly selected from one of the eight conditions for a total of 333 trials with varying dollar amounts across trials. Importantly, to maintain independence of choices (e.g., no carry-over effects from previous trials), participants were not shown the outcome of any trial. Instead, at the end of the experiment, one trial was randomly selected, and the choice the participant made on that trial resulted in a monetary outcome that affected their payment. ***Figure 2*** depicts an example from each condition in Study 2 (high stakes). The amount that could be gained/lost on a given trial was 20x in Study 2 compared to Study 1; this is true for both the unambiguous and ambiguous dollar values. The unambiguous dollar values for each condition varied based on pre-determined values (see Supplemental Materials Figure SM1 for matrices of values).

### Gambling Propensity – High (vs Low) Stakes Decreases Gambling in Most Conditions but Increases Gambling with Ambiguous Sure Loss

**Figure 3:**
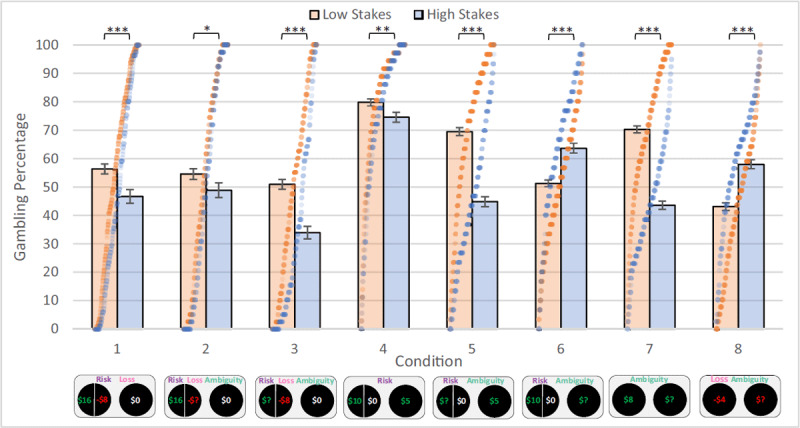
Gambling Propensity Per Condition in High vs Low Stakes. Bars represent mean gambling percentage for high and low stakes per condition (error bars are standard error). “Gambling” refers to choosing the risky 50%/50% option in Conditions 1–6 or the ambiguous option in Conditions 7–8. Dots indicate individual data points; they are arranged in ascending order within each condition per an empirical cumulative distribution function. Effects of low vs high stakes are significant within each condition. Below X-axis is an example of a trial from each condition; “Risk,” “Loss,” and “Ambiguity” indicate whether each parameter type is present in that condition. All significant differences pass Holm-Bonferroni cutoffs for multiple comparisons.

See ***Figure 3*** for gambling propensity. “Gambling” is defined as choosing the 50%/50% risky option in Conditions 1–6 and choosing the ambiguous option in Conditions 7–8. Overall, participants showed significantly less gambling with high compared to low stakes in Conditions 1–5 and 7: Condition 1 (t(209) = 4.872, p < .001, Holm-Bonferroni cutoff = .013, d = .336, 95% CI: –15.582, –6.605), 2 (t(209) = 2.508, p = .013, Holm-Bonferroni cutoff = .050, d = .173, 95% CI), 3 (t(209) = 7.656, p < .001, Holm-Bonferroni cutoff = .017, d =.528, 95% CI: –22.062, –13.027), 4 (t(209) = 3.110, p = .002, Holm-Bonferroni cutoff = .025, d = .215, 95% CI: –8.839, –1.981), 5 (t(209) = 14.875, p < .001, Holm-Bonferroni cutoff = .007, d = 1.026, 95% CI: –28.493, –21.825), and 7 (t(209) = 16.756, p < .001, Holm-Bonferroni cutoff = .006, d = 1.156, 95% CI: –30.405, –24.003). Conversely, participants showed significantly more gambling with high compared to low stakes in Condition 8 (t(209) = –8.288, p < .001, Holm-Bonferroni cutoff = .008, d = .572, 95% CI: 10.497, 17.049) (this was the only condition with no possible gains), as well as an increased preference for unambiguous risky gains over ambiguous sure gains with high vs low stakes in Condition 6 (t(209) = –7.087, p < .001, Holm-Bonferroni cutoff = .010, d = .489, 95% CI: 9.235, 16.352).

### Model Selection – Model 5 (Prospect Theory Model with Additional Ambiguity Parameters for Ambiguous Sure Gains, Ambiguous Risky Gains, Ambiguous Sure Losses, and Ambiguous Risky Losses) Is Winning Model with Both Low and High Stakes

Because most conditions contain more than one decision-making construct of interest (i.e., risk, gain/loss, or ambiguity), it is difficult to know what is driving decision-making in each condition simply by looking at participants’ behavioral gambling propensity. To investigate the underlying motivations for decision-making, we used computational modeling to estimate parameters (i.e., risk preference, loss aversion, ambiguity preference/aversion) and their relative contributions to behavior in five versions of the prospect theory model. Model 1 was the traditional prospect theory model (risk preference and loss aversion), and Models 2–5 were variations of the traditional prospect theory model with ambiguity preference/aversion parameters of increasing complexity across models.

See ***Table 1*** for model comparison. In Studies 1 and 2, the Traditional Prospect Theory model (Model 1) performed better than both null models, and all ambiguity models (Models 2–5) performed better than the Traditional Prospect Theory model. This suggests that including ambiguity parameter(s) improved model fit beyond parameterizing just risk and loss. In Studies 1 and 2, Model 5 (choice consistency, risk preference, loss aversion, and four ambiguity parameters: ambiguous risky loss, ambiguous sure loss, ambiguous risky gain, and ambiguous sure gain) was the best-fitting model as assessed by AIC (lower is better), R^2^, and out-of-sample accuracy. Thus, the remaining analyses focus on Model 5. Additionally, R^2^ and out-of-sample accuracy improved from Study 1 (low stakes) to Study 2 (high stakes), suggesting the model is more accurate with increasing stakes.

**Table 1:** Model predictive accuracy was calculated in two out-of-sample ways. In the within-subjects analysis, model parameters were estimated for each subject using approximately 5/6 of their data and used to test accuracy in the remaining trials. In the between-subjects analysis, group model parameters are estimated in 29/30 participants and used to test accuracy in the remaining subjects. Note that for Null Models 1 and 2, the accuracy is calculated in-sample since the models have no parameters. Model 5 was the best-fitting model in both studies, and model fit improved with higher stakes.


(A) MODEL COMPARISON (STUDY 1: LOW STAKES $3.60–$5.70)					

MODEL DESCRIPTION	NUMBER OF PARAMETERS	R^2^	AIC	MODEL ACCURACY: WITHIN-SUBJECTS OUT-OF-SAMPLE	MODEL ACCURACY: BETWEEN-SUBJECTS OUT-OF-SAMPLE

Null Model 1: 50% Probability to Gamble on Each Trial	0	.000	460.4	50.0%	

Null Model 2: Average Gambling Rate for Given Participant on Each Trial	0	.162	385.9	69.4%	

Model 1: Traditional Prospect Theory	3	.334	312.6	68.9%	61.9%

Model 2: General Ambiguity	4	.412	278.6	72.8%	63.1%

Model 3: Ambiguous Gains and Losses	5	.441	267.2	74.3%	63.2%

Model 4: Ambiguous Loss or No-Loss Contexts	5	.445	265.3	74.5%	63.3%

Model 5: Ambiguous Sure/Risky Gains/Losses	7	.473	256.4	75.6%	65.1%

**(B) MODEL COMPARISON (STUDY 2: HIGH STAKES $6–$48)**					

**MODEL DESCRIPTION**	**NUMBER OF PARAMETERS**	**R^2^**	**AIC**	**MODEL ACCURACY: WITHIN-SUBJECTS OUT-OF-SAMPLE**	**MODEL ACCURACY: BETWEEN-SUBJECTS OUT-OF-SAMPLE**

Null Model 1: 50% Probability to Gamble on Each Trial	0	.000	460.8	50.0%	

Null Model 2: Average Gambling Rate for Given Participant on Each Trial	0	.145	393.9	68.3%	

Model 1: Traditional Prospect Theory	3	.416	275.2	73.1%	65.6%

Model 2: General Ambiguity	4	.496	240.2	77.1%	66.7%

Model 3: Ambiguous Gains and Losses	5	.529	227.2	78.6%	67.0%

Model 4: Ambiguous Loss or No-Loss Contexts	5	.522	230.4	78.3%	66.7%

Model 5: Ambiguous Sure/Risky Gains/Losses	7	.552	220.5	79.5%	67.7%


### Model 5 – Excellent Recovery of Model Parameters and Correlation Between Real and Model-Recovered Gambling Rates

**Figure 4:**
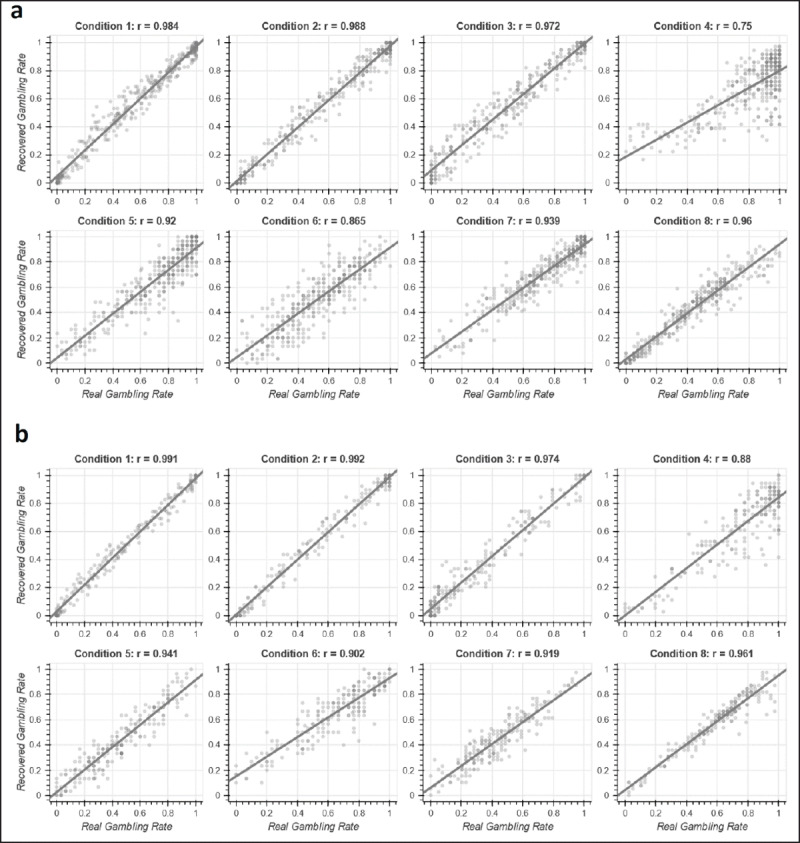
Model 5 Real Gambling Rate vs Model-Recovered Gambling Rate. Figure shows correlations within each condition for Model 5 between real gambling rate and model-recovered gambling rate. Results show our model was very accurate in its prediction of real gambling rate, suggesting the model’s validity. Panel **a** is Study 1 (low stakes: $3.60–$5.70), and panel **b** is Study 2 (high stakes: $6–$48).

Within Model 5, we correlated participants’ real gambling rate with the model-recovered gambling rate for all eight conditions. See ***Figure 4*** for details. In Study 1, most conditions had nearly perfect correlations (rs > .920), and Condition 4 had a high correlation (r = .750). In Study 2, most conditions again had nearly perfect correlations (rs > .902), and Condition 4 had a very high correlation (r = .880). Additionally, we correlated our model’s estimated parameters and model-recovered parameters, which were very high in low stakes (mean r = .969, range .926 to .987) and high stakes (mean r = .973, range .897 to .996). Thus, our model was able to accurately predict gambling rate and recover parameters, confirming its validity.

### Model 5 – Greater Monetary Stakes Result in Greater Aversion to Risk and Ambiguous Gains but Greater Preference for Ambiguous Sure LossES

**Figure 5:**
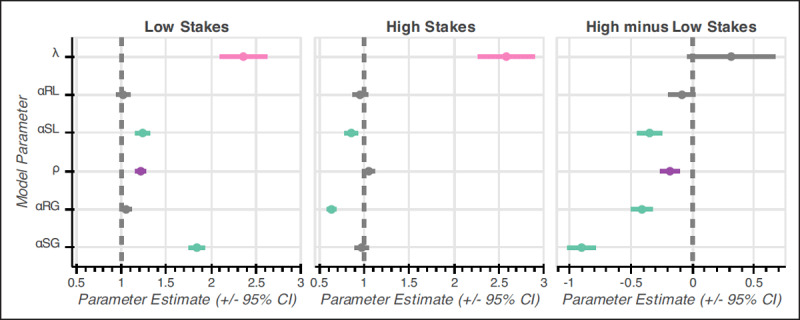
Model 5 Parameter Results – High and Low Stakes. Figure shows point estimates of parameters and 95% confidence intervals. Values that are significant are in color (all significant results passed Holm-Bonferroni correction); null results are in gray. Study 1 was low stakes ($3.60–$5.70; N = 367), and Study 2 was high stakes ($6–$48; N = 210). λ = Loss Aversion, ρ = Risk Preference, αRL = Ambiguous Risky Loss Aversion, αSL = Ambiguous Sure Loss Aversion, αRG = Ambiguous Risky Gain Preference, and αSG = Ambiguous Sure Gain Preference. For “Aversion” parameters (i.e., λ, αRL, αSL), greater values indicate greater aversion. For “Preference” parameters (e.g., ρ, αRG, αSG), greater values indicate greater preference.

See ***Figure 5*** for summary of results and Supplemental Materials Table SM5 for details on statistical results. For “preference” parameters (e.g., risk preference), values greater than 1 indicate preference, and values less than 1 indicate aversion. For “aversion” parameters (e.g., loss aversion), values greater than 1 indicate aversion, and values less than 1 indicate preference.

Within the loss domain, results show that with low and high stakes, participants exhibited (unambiguous) loss aversion (ps < .001) with no significant difference between low and high stakes (p = .084). Participants showed no ambiguous risky loss preference/aversion with low or high stakes (ps > .278). Conversely, participants showed ambiguous sure loss aversion with low stakes and ambiguous sure loss preference with high stakes (ps < .001) with a significantly greater preference for ambiguous sure losses with high vs low stakes (p < .001).

Within the gain domain, participants exhibited (unambiguous) risk preference with low stakes (p < .001), no risk preference/aversion with high stakes (p = .173), and significantly lower risk preference with high vs low stakes (p < .001). Participants showed no ambiguous risky gain preference/aversion with low stakes (p = .122) but showed aversion with high stakes (p < .001), and there was a significant increase in ambiguous risky gain aversion from low to high stakes (p < .001). Additionally, participants showed ambiguous sure gain preference with low stakes (p < .001) but no preference/aversion with high stakes (p = .471), and there was a significant decrease in ambiguous sure gain preference from low to high stakes (p < .001). Lastly, when directly comparing unambiguous risky gains and ambiguous sure gains within a choice (i.e., Condition 6), participants showed no preference for either option with low stakes (p = .301) but showed preference for unambiguous risky gains over ambiguous sure gains with high stakes (p < .001); this included a significant increase in unambiguous risky gain preference from low to high stakes (p < .001).

**Figure 6:**
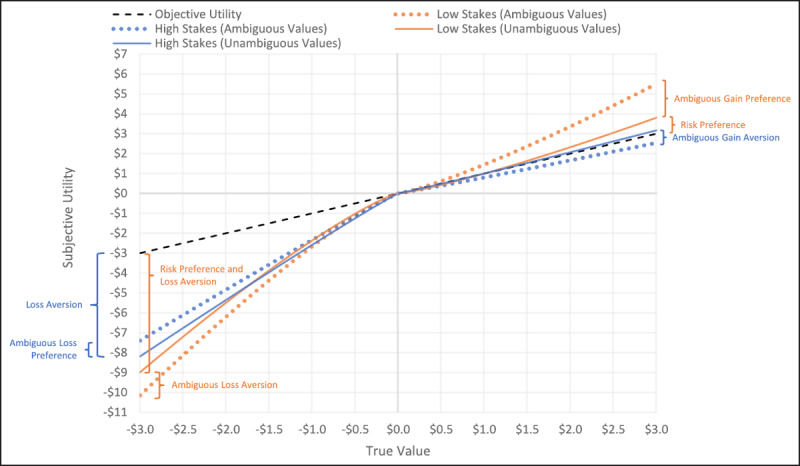
Low and High Stakes Results – Prospect Theory Model with Ambiguous Outcome Magnitudes. This figure shows the true parameter values derived from the best-fitting model (Model 5) in Study 1 (Low Stakes; $3.60–$5.70) and Study 2 (High Stakes; $6–$48). For concision, we collapsed the ambiguity parameters into two parameters: ambiguous gain preference/aversion and ambiguous loss preference/aversion. This was done within each study by averaging the a) ambiguous risky gain and ambiguous sure gain parameters and b) ambiguous risky loss and ambiguous sure loss parameters using the actual mean parameter values derived in each study. Parameter values were as follows: Low Stakes (ρ = 1.217, α_G_ = 1.447, λ = 2.361, α_L_ = 1.129) and High Stakes (ρ = 1.050, α_G_ = .803, λ = 2.258, α_L_ = .903).

Overall, with increasing stakes, there was increased aversion to risky and sure ambiguous gains, unambiguous risk, and a relatively greater aversion to ambiguous sure gains over unambiguous risky gains (Condition 6); conversely, there was aversion for ambiguous sure losses with low stakes and preference for ambiguous sure losses with high stakes. These effects are visualized in ***Figure 6***.

### Emotionality – Wide Distribution of Trait Anxiety and Trait Depression with Many Participants Who Likely Have Clinically Severe Anxiety And/Or Depression

We pre-registered our calculations of trait anxiety and trait depression into composite scores. To get a robust measure of each, we combined the anxiety subscales and depression subscales of four measures (DASS-21, OASIS, PANAS, STAI; see Methods) and put them on to a 0–1 scale, where a value of 0 indicates endorsing no anxiety/depression on any item on any scale (25 items for anxiety, 35 items for depression), and a value of 1 indicates endorsing maximum anxiety/depression on all items on all scales. Thus, scores of 0 and 1 indicate extremely low or extremely high anxiety/depression, respectively.

**Figure 7:**
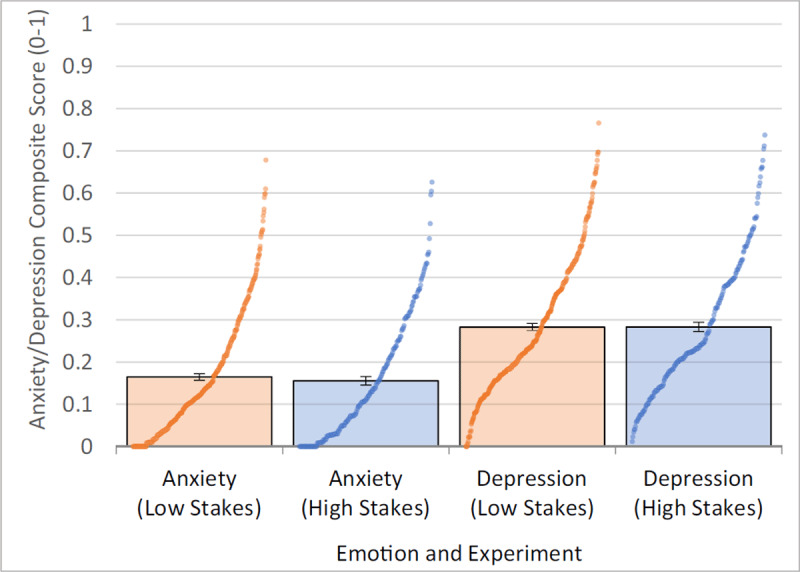
Trait Anxiety and Depression. Figure shows a bar plot of means with standard error and an empirical cumulative distribution function, where individual scores are plotted as dots. Figure shows a wide and consistent distribution of trait anxiety and trait depression in both the low stakes and high stakes studies.

Results show that trait anxiety ranged from 0 to .678 (Study 1: M = .165, SD = .149; Study 2: M = .155, SD = .147), and trait depression ranged from 0 to .766 (Study 1: M = .282, SD = .163; Study 2: M = .283, SD = .163), suggesting scores ranged from very low to very high (***Figure 7***). Additionally, looking at the DASS-21 Anxiety subscale and the OASIS, 26.98% of participants in Study 1 had at least moderate anxiety with the DASS-21 (score of 10+), and 37.87% met criteria for likely having an anxiety disorder (score of 8+; Campbell-Sills et al., 2009). While we did not use a scale with a clinical cutoff for depression, the DASS-21 Depression subscale similarly showed 29.43% of participants had at least moderate depression (score of 14+). Similar results were found in Study 2: DASS-21 Anxiety (25.71%), OASIS (30.95%), and DASS-21 Depression (31.43%). Additionally, participants had greater depression than anxiety scores in both studies (ps < .001). In total, this suggests that our sample distribution of trait anxiety and trait depression was wide and likely contained many individuals who met clinical criteria for an anxiety or depressive disorder. Furthermore, the correlations between trait anxiety from Study 1 to Study 2 (r = .909) and trait depression from Study 1 to Study 2 (r = .932) were both very strong, and their means and distributions were very similar across studies. Lastly, we assessed within each study whether trait anxiety and trait depression were multicollinear using variance inflation factor (VIF) (Thompson et al., 2017), where VIF scores > 10 may indicate multicollinearity. Results showed that there were no multicollinearity concerns in Study 1 (VIF = 2.53) or Study 2 (VIF = 2.75).

### Model 5 Emotionality Results – No Significant Association of Trait Anxiety or Trait Depression with Risk, Loss, or Ambiguity Preference/Aversion

**Figure 8:**
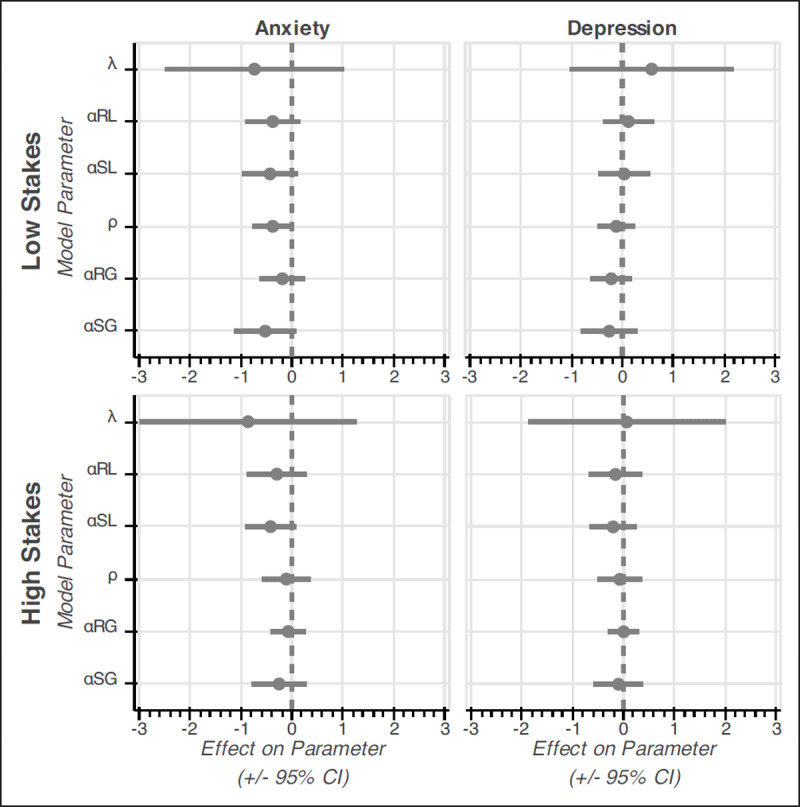
Model 5 Anxiety and Depression Results. Figure shows effects of anxiety and depression on model parameters and 95% confidence intervals. All results are null and therefore in gray. Study 1 was low stakes ($3.60–$5.70), and Study 2 was high stakes ($6–$48). λ = Loss Aversion, ρ = Risk Preference, αRL = Ambiguous Risky Loss Aversion, αSL = Ambiguous Sure Loss Aversion, αRG = Ambiguous Risky Gain Preference, and αSG = Ambiguous Sure Gain Preference. For “Aversion” parameters (i.e., λ, αRL, αSL), greater values indicate greater aversion. For “Preference” parameters (e.g., ρ, αRG, αSG), greater values indicate greater preference.

We used trait anxiety and trait depression from Study 1 and Study 2 to predict model parameters in Study 1 and Study 2, respectively. Results of trait anxiety (ps > .068) and trait depression (ps > .294) showed no significant effects on any model parameter (***Figure 8***) or on Condition 6 gambling rate (see Supplementary Materials Tables SM6–9 for statistical details).

This absence of association between anxiety or depression and model parameters was confirmed by Bayesian regression analyses, which allowed us to determine the degree to which the null hypothesis was supported over the experimental hypothesis. Our Bayes factor results showed that the effects of trait anxiety and trait depression on model parameters in Studies 1 and 2 were always in favor of the null hypothesis (BF_null_ range 1.952 to 6.655, mean = 5.254; see Supplementary Materials Table SM10 for details), suggesting that the odds of the null hypothesis being true was ~5.25x greater than the odds of the experimental hypothesis being true.

Lastly, one possibility for the absence of an effect of trait anxiety or depression on model parameters is that many participants may not have paid attention when answering the questionnaires, thus leading to noisy results. To assess the validity of participants’ responses, we used the R package “Careless” (Yentes & Wilhelm, 2021), which detects whether participants responded to questionnaire items without regard to their content. Specifically, within each study, we investigated intra-individual response variability scores, psychometric antonym scores, psychometric synonym/antonym scores, and split-half reliability scores. We calculated participants’ averages for each Careless measure and created Z-scores. If participants had Z-scores ≤ –2 (intra-individual response variability, synonym, split-half reliability) or ≥ 2 (synonym/antonym), we removed them from analyses since these indicate potentially “careless” responding. Very few participants met these criteria for careless responding (low stakes: intra-individual response variability (0 participants), split-half reliability (11), synonym (11), antonym (2); high stakes: intra-individual response variability (1 participant), split-half reliability (11), synonym (5), antonym (0)). There was a total of 19/367 and 15/210 unique participants within each study who exceeded these Z-scores and were dropped from analyses. This is also in line with the finding reported above that anxiety and depression scores were highly consistent across studies, suggesting that participants overall paid attention to the questionnaires. Importantly, the effects of trait anxiety (ps > .093) and trait depression (ps > .326) on model parameters were unaffected by the exclusion of these participants. Taking into consideration our frequentist, Bayesian, and “Careless” analyses above, our results suggest that trait anxiety and trait depression had no association with risk preference, loss aversion, and ambiguity preference/aversion.

## Discussion

We report two studies investigating economic decision making with risk, loss, and ambiguous outcome magnitude. Study 1 involved low stakes ($3.60–$5.70), and Study 2 involved high stakes ($6–$48). We conducted computational modeling to determine whether including ambiguity parameters would improve model fit over and above traditional prospect theory, which is a seminal model that parameterizes risk preference and loss aversion. We additionally investigated participants’ preferences/aversions towards ambiguous outcome magnitudes for risky and sure gains and losses. Lastly, we investigated the association between trait anxiety and trait depression and economic decision-making.

In summary, our results showed that including ambiguity parameters to traditional risk preference and loss aversion parameters improved model fit. Interestingly, increasing stakes increased risk aversion, ambiguous risky gain aversion, ambiguous sure gain aversion, and ambiguous sure loss preference. However, we found no association between trait anxiety or trait depression and economic decision-making. This was supported by Bayesian analyses showing ~5.25x odds that trait anxiety and trait depression had no effect vs having an effect.

To elaborate on our results: in the loss domain, participants exhibited similar levels of (unambiguous) loss aversion regardless of low or high stakes, though there was a trend towards greater loss aversion with high stakes. Additionally, with both low and high stakes, there was no preference/aversion for ambiguous risky loss. However, participants showed opposite tendencies with ambiguous sure loss depending on stakes: low stakes led to ambiguous sure loss aversion, whereas high stakes led to ambiguous sure loss preference. This is consistent with limited prior research showing that individuals have a preference for ambiguous vs unambiguous losses (Ho et al., 2002), though ours expands upon this by showing that ambiguous aversion/preference depends on low vs high stakes and has no effect with ambiguous risky loss.

Furthermore, our results within the gain domain showed that participants exhibited (unambiguous) risk preference with low stakes and no preference/aversion with high stakes; this included a significant decrease in risk preference from low to high stakes. This is consistent with previous studies demonstrating increased risk aversion with increased stakes (Binswanger, 1980; Fehr-Duda et al., 2010; Harrison et al., 2005; Holt & Laury, 2002, 2005; Kachelmeier & Shehata, 1992; Markowitz, 1952), but neither of our studies demonstrated a true aversion to unambiguous risk. Unlike our studies, other studies demonstrating risk aversion used only unambiguous outcome magnitudes in their experimental designs, which might have led to risky conditions feeling relatively more uncertain compared to non-risky conditions in their experiments. Indeed, previous work on ambiguous likelihoods showed that individuals are averse to ambiguous vs unambiguous likelihoods (Camerer & Weber, 1992; FeldmanHall et al., 2016; Fox & Tversky, 1995; Heath & Tversky, 1991; Huettel et al., 2006; Ruderman et al., 2016). In our case, perhaps unambiguous risk provided relatively greater perceived competence (Fox & Tversky, 1995) than ambiguous outcome magnitudes, leading to greater risk preference. This notion is supported in part by our finding with high stakes that individuals preferred an unambiguous risky gain over an ambiguous sure gain within a single choice (Condition 6).

Interestingly, high stakes led to greater aversion of ambiguous sure and risky gains. Specifically, low stakes led to a preference for ambiguous sure gains and no preference/aversion for ambiguous risky gains; high stakes showed a reduction in preference for both of these, where ambiguous sure gains now showed no preference/aversion, and ambiguous risky gains showed aversion. In short, it seems that higher stakes increase ambiguous gain aversion. Thus, examining these results in total, it appears our high stakes results are consistent with previous studies showing risk aversion (Binswanger, 1980; Fehr-Duda et al., 2010; Harrison et al., 2005; Holt & Laury, 2002, 2005; Kachelmeier & Shehata, 1992; Markowitz, 1952), aversion to ambiguous gains (González-Vallejo et al., 1996; Kuhn & Budescu, 1996; Oliver, 1972), and preference for ambiguous losses (Ho et al., 2002). Our experiments add to this literature by showing that increasing stakes a) increases ambiguous sure and risky gain aversion and b) increases ambiguous sure loss preference.

Additionally, our computational modeling results showed that the traditional prospect model theory (which models unambiguous risk preference and loss aversion) was not as accurate as our ambiguity models that included risk, loss, and at least one ambiguity parameter. Our best-fitting model in both studies included separate parameters for ambiguous risky gains, sure gains, risky losses, and sure losses, and this model fit the best even when accounting for parsimony via the AIC. Because all of our ambiguity models outperformed the unambiguous traditional prospect theory model, this may suggest that in order to expand prospect theory to account for ambiguous outcome magnitudes, ambiguity must be modeled. Furthermore, the inspiration to model ambiguous risky gains, sure gains, risky losses, and sure losses separately stemmed from the intuition that people may treat these types of decisions differently (e.g., is a sure ambiguous gain treated differently than a risky ambiguous gain?). Our computational modeling suggests that this was the case. It would be prudent, though, to highlight the question of how or to what degree ambiguous gains and losses should be divided into risky vs sure parameters as in our winning model. For example, our experiment defined risk as a 50%/50% choice and a sure choice as having 100% of occurring. However, would our ambiguous risky gain and ambiguous risky loss parameters apply to a 25%/75% choice, as well, or would new parameters be needed? Psychologically, there is likely an intrinsic difference between certain and uncertain situations, but the degree to which ambiguity interacts with different probabilities is an empirical question. Thus, future studies could incorporate ambiguous outcome magnitudes with several probabilities (e.g., 50%/50%, 25%/75%, 100%). Along a similar line, our experiment manipulated unambiguous vs ambiguous outcome magnitudes, but we utilized only unambiguous probabilities since our goal was to focus on ambiguous outcome magnitude. Future work could assess the interactions between ambiguous outcome magnitudes and ambiguous likelihoods within one experiment. This could help us better understand the potential interactions between unambiguous/ambiguous magnitudes/likelihoods and more closely approximate real-world decision-making.

Furthermore, contrary to hypotheses, trait anxiety and depression were not significantly associated with preference for or aversion to risk, loss, or ambiguity. These null effects occurred regardless of whether we used our full samples or excluded the relatively few individuals who showed “careless” responding to the questionnaires (Yentes & Wilhelm, 2021). These findings were confirmed by Bayesian regression analyses showing that the null hypothesis (in which trait anxiety and trait depression are not associated with model parameters) was always better-supported by the data than the experimental hypothesis (in which trait anxiety and trait depression are associated with model parameters) by an average factor of 5.25x, showing moderate support of the null hypothesis. Our experiments thus add to the mixed results seen in previous studies regarding unambiguous risk and loss (Baek et al., 2017; Chandrasekhar Pammi et al., 2015; Chapman et al., 2007; Charpentier et al., 2017; Ernst et al., 2004; Harpaz-Rotem et al., 2017; Lauriola & Levin, 2001; Leahy et al., 2012; Lerner & Keltner, 2000, 2001; Maner et al., 2007; Raghunathan & Pham, 1999; Sip et al., 2018; Smoski et al., 2008). It is unclear why we did not observe any association between trait anxiety or trait depression on economic decision-making parameters. One possibility is that previous studies have sometimes used clinical samples (Chapman et al., 2007; Charpentier et al., 2017; Sip et al., 2018), whereas we did not recruit based on clinical disorder. However, this is unlikely to be the reason for this discrepancy since results from our study show that ~30–38% of participants likely had an anxiety disorder, and ~25–31% of participants had at least moderate trait anxiety or trait depression. While we did not have a clinical cutoff in our questionnaires for trait depression, participants had a higher depression score than anxiety, suggesting that we may have had similar or greater clinical depression rates. Thus, we had a wide distribution of trait anxiety and trait depression that likely led to a valid assessment of these traits at both clinical and non-clinical levels. Given the mixed results of previous studies, perhaps our experiments are at an advantage due to their large sample size (Study 1: N = 367; Study 2: N = 210), assessment of both low and high stakes (Study 1: $3.60–$5.70; Study 2: $6–$48), large number of trials per condition (333 trials across 8 conditions), and because participant decisions affected their final payment (i.e., these were not hypothetical decisions). Moreover, while our findings suggest trait anxiety and trait depression are not related to economic decision making, this does not necessarily mean they are unrelated to other forms of decision making. Indeed, it is very common in therapy for a clinically anxious or depressed individual to overestimate the likelihood or magnitude that negative outcomes will occur (or, underestimate positive outcomes; e.g., catastrophizing; Beck et al., 1979; Beck, 1967; Beck et al., 1974; Craske et al., 2014). Perhaps our null results suggest that trait anxiety and trait depression are too broad of constructs to measure economic decision-making tendencies and that narrower traits/constructs are needed; perhaps anxiety or depression related to finances would be a more appropriate predictor of economic decision making. Alternatively, economic decision-making may be too narrow of a task to assess general decision-making tendencies; perhaps domain-specific anxiety or depression (e.g., social anxiety) would be more predictive of risk, loss, or ambiguity aversion in a domain-specific decision-making task (e.g., with social outcomes). Thus, future work could either assess trait anxiety and depression related to finances or match the anxiety and depression domain more closely with the type of decision-making task.

Lastly, our experiments have a few limitations. First, our high stakes study (N = 210) had a lower sample size than our low stakes study (N = 367); however, both sample sizes are large and well-powered, especially given that they were within-subjects (i.e., same participants in Study 2 as in Study 1), and the results were consistent when using the full sample from the low stakes study or using the 210 participants that were in both studies (see Supplementary Materials Table SM11). Second, our low stakes study was conducted approximately one year prior to our high stakes study. This one-year gap likely prevented any practice effects or order effects, but a better approach would have been to counterbalance the order in which each study occurred.

In conclusion, our studies expand upon risk/no-risk and gains/losses as variables that drive economic decision-making by including an additional variable: unambiguous vs ambiguous outcome magnitude. We also add to the “ambiguity” economic decision-making literature, which is fairly scarce and has largely focused on ambiguous outcome likelihoods rather than ambiguous outcome magnitudes. Including ambiguity parameters into the traditional prospect theory model improved fit and accuracy of the model, suggesting that ambiguous outcome magnitude is a separable construct from risk and loss. Our studies also showed that increasing stakes increased aversion to risk, ambiguous sure gains, and ambiguous risky gains, but it increased preference for ambiguous losses. Overall, whether individuals have an aversion to ambiguous outcome magnitudes depends on whether a) the stakes are high or low, b) the ambiguous outcomes are gains or losses, and c) the ambiguous loss is risky or sure. The overall tendency was to be averse to high stakes ambiguous gains and low stakes ambiguous sure losses. Lastly, there was no detectable association between trait anxiety and depression and economic decision-making related to risk, loss, or ambiguity. This may suggest that anxiety and depression are not related to economic decision-making or that the emotional constructs and task need to be more closely matched to one another.

## Methods

### Participants

To efficiently acquire a nation-wide community sample, we used the online platform Amazon Mechanical Turk (i.e., MTurk; Hauser et al., 2019). MTurk has been shown to provide valid data (Casler et al., 2013; Hauser et al., 2019), and we enforced several stringent quality assurance criteria to maximize high quality data (see Supplemental Materials). Study 1 had low monetary stakes ($3.60–$5.70); Study 2 had high monetary stakes ($6–$48). Participants in Study 2 (N = 210) also participated in Study 1 (N = 367), making this a within-subjects design. Study 1 was completed approximately one year prior to Study 2. In Study 1, participants were 50.95% male, 47.14% female, 0.82% male-to-female transgender, 0.54% gender-fluid, and 0.54% chose not to answer; mean age 39.45 years (SD = 18.52); and 5.72% Asian, 6.27% Black or African-American; 4.36% Hispanic or Latinx, 77.66% White, and 5.99% Multiracial. In Study 2, participants were 52.38% male, 46.67% female, 0.48% male-to-female transgender, and 0.48% agender; mean age 43.11 years (SD = 37.20); and 7.14% Asian, 3.81% Black or African-American; 2.86% Hispanic or Latinx, 81.43% White, and 4.76% Multiracial. These studies were approved by the California Institute of Technology Institutional Review Board (#18–0867), and all participants provided informed consent using our online survey prior to commencing the study.

### Materials and Apparatus

We used MTurk to collect online data and recruit participants; Qualtrics to conduct informed consent and gather most self-report questionnaire data; and Pavlovia to host our PsychoPy 3.0.5 gambling experiment. MTurk included links to our Qualtrics and Pavlovia websites.

Gambling stimuli included two pairs of circles representing the left and right choices (see ***Figure 2***). There were three versions of these choice pairs: a) Left (50%/50%), Right (100%), b) Left (100%), Right (50%/50%), and c) Left (100%), Right (100%). “50%/50%” indicates a 50% chance of receiving either of the two outcomes for that choice and was represented by a circle with a vertical line splitting it in half, and “100%” indicates a 100% chance of receiving that outcome and was represented by a circle. Gain amounts were color-coded as green, loss amounts as red, and $0 as white. Ambiguous outcome magnitudes were represented as “$?” or “–$?” and color-coded as green or red to represent gains or losses, respectively. See Supplemental Materials for details on trial sequence and counterbalancing.

### Measures – Behavioral

We recorded the binary Gamble/No Gamble choices participants made per trial. We additionally computed percentage of gambling and not gambling (Conditions 1–6) and choosing the ambiguous sure option or the unambiguous sure option (Conditions 7 and 8) per Condition. Trials in which no choice was made were excluded from percentage calculation. Throughout the paper, “gambling” refers to choosing the risky 50%/50% option (Conditions 1–6), or, in the cases where both options were 100%, “gambling” refers to choosing the ambiguous option (Conditions 7–8).

### Measures – Self-Report

*Depression, Anxiety, and Stress Scale (DASS-21; Antony, et al., 1998; Lovibond & Lovibond, 1995)*. The DASS-21 measures severity of depression, anxiety, and stress and is designed to maximize the discriminative measurement of depression and anxiety (e.g., by excluding items with symptom overlap).

*Overall Anxiety Severity and Impairment Scale (OASIS; Norman et al., 2006)*. The OASIS transdiagnostically measures frequency and severity of anxiety, as well as functional impairment due to anxiety. A cutoff of ≥ 8 has been shown to indicate individuals who likely meet criteria for an anxiety disorder (Campbell-Sills et al., 2009).

*Positive and Negative Affect Schedule (PANAS; Watson & Clark, 1999)*. The PANAS measures general positive affect (PA) and general negative affect (NA). We also included the Fear, Sadness, and Hostility subscales to measure fear/anxiety, sadness/depression, and hostility/anger. In total, we used 31 items from the PANAS. For each, we measured trait affect (“in general, that is, on the average”).

*State-Trait Anxiety Inventory – Trait Version (STAI; Spielberger & Gorsuch, 1983)*. Although initially considered a measure of just anxiety (Spielberger & Gorsuch, 1983), the STAI was re-evaluated and determined to have separate depression and anxiety subscales (Bieling et al., 1998). The STAI Anxiety subscale is moderately correlated with DASS-21 Anxiety (r = 0.55) and Depression (r = 0.53). The STAI Depression subscale is strongly correlated with DASS-21 Depression (r = 0.64) and modestly correlated with DASS-21 Anxiety (r = 0.36). The STAI Total score is strongly positively correlated with DASS-21 Depression (r = 0.67) and moderately correlated with the DASS-21 Anxiety (r = 0.47).

We pre-registered the calculation of our composite scores for trait anxiety and trait depression based on the above questionnaires, which were previously shown to measure anxiety and depression. We used a composite score to provide a more robust measurement of trait anxiety and depression (Zinbarg et al., 2016). The questionnaires used in trait anxiety composite score were the STAI Anxiety subscale, DASS-21 Anxiety subscale, PANAS Fear subscale, and OASIS. The questionnaires used in the trait depression composite score were the STAI Depression subscale, DASS-21 Depression subscale, PANAS Sadness subscale, and PANAS Positive Affect subscale (reverse-coded). See Supplementary Materials Tables SM1 and SM2 for details on the questionnaires, items used in the composite score, and specific calculations.

### Procedure

Participants who selected our study on MTurk opened our Qualtrics page and completed informed consent. Participants then completed a demographics questionnaire, all trait self-report questionnaires, and the one-item colorblindness test. Participants subsequently started the economic decision-making experiment by reading instructions for the gambling experiment and completing six multiple choice quiz items to assess their understanding of the experiment’s instructions (i.e., factual manipulation check; Goodman et al., 2013; Litman et al., 2015). Importantly, participants were *not* explicitly informed what the range of possible ambiguous outcome values was (i.e., “$?” or “–$?”) in order to facilitate individual differences in estimation of the ambiguous amounts. Then, participants completed 33 practice trials, the state Positive and Negative Affect Schedule (Watson & Clark, 1999), and the 333 experimental trials. Afterwards, participants were informed of their final monetary compensation. In Study 1, payment was $4.50 plus the outcome of that trial, with final payment ranging from $3.60 to $5.70; in Study 2, payment was $24 plus the outcome of the trial, with final payment ranging from $6 to $48. Thus, the amount that could be gained/lost was –$0.90 to $1.20 in Study 1 and –$18 to $24 in Study 2, which differed between studies by a factor of 20. Participants then completed free-response questions about the experiment (e.g., describe any problems/feedback regarding the experiment), were debriefed, and paid.

### Data Analysis

We used MATLAB R2018a (The MathWorks, Inc., Natick, MA) to conduct computational modeling of the behavioral data, Stata/MP2 Version 15.1 (StataCorp, College Station, TX) to conduct inferential statistical tests, JASP 0.14.1.0 for Bayesian analyses, and R 4.0.2 for “Careless” analyses.

### Computational Modeling

For computational modeling, we estimated risk aversion and loss aversion for each participant using a traditional three-parameter prospect theory model (Model 1) (Kahneman & Tversky, 2013; Sokol-Hessner et al., 2009, 2013; Tversky & Kahneman, 1992). As a novel aspect of our report, we also included multiplicative ambiguous outcome magnitude parameters (i.e., “ambiguity parameters”) to the traditional prospect theory model in four additional models. These models included: Model 2 – one general ambiguity parameter (includes all six ambiguity Conditions); Model 3 – separate ambiguous gain (Conditions 3, 5–7) and ambiguous loss (Conditions 2, 8) parameters; Model 4 – loss context (Conditions 2, 3, 8) and no-loss context (Conditions 5–7) ambiguity parameters; and Model 5 – ambiguous risky gain (Conditions 3, 5), risky loss (Condition 2), sure gain (Conditions 6–7), and sure loss (Condition 8) parameters. All of these models were compared to two null models in which there was a 50% gambling rate (Null Model 1) or the participant-specific gambling rate (Null Model 2) without any parameters. We then evaluated the models based on pseudo-R^2^_,_ Akaike Information Criterion (AIC) (Burnham & Anderson, 2004), and out-of-sample model accuracy. Higher pseudo-R^2^ indicates the proportion of variance in the data explained by the model, and lower AIC scores indicate better model fit. Out-of-sample model accuracy was calculated in two ways: within- and between-subjects. For within-subjects accuracy, gambling data from each subject was split into six groups of trials with equal proportion of Conditions in each group. Model parameters were estimated from five groups of trials, and accuracy was tested on the remaining group. This process was repeated for each group, then 100 times with a different assignment of trials to groups. For between-subjects accuracy, subjects were split into nine groups of six participants each. Model parameters were estimated from all subjects from eight groups, then mean parameter estimates were used to predict choice accuracy in the remaining group. This process was repeated for each group, then 100 times with a different assignment of subjects to groups.

Equations below (***Table 2***) are representative of the winning model, Model 5, which contains four ambiguity parameters estimated separately for risky gains (Conditions 3 and 5), sure gains (Conditions 6 and 7), risky losses (Condition 2), and sure losses (Condition 8). Ambiguity parameters were implemented as a multiplicative weight to the mean rational value of ambiguous amounts in each condition. Specifically, for Conditions 1–6, we used the unambiguous values from other conditions that were structurally identical to the condition of interest, except the other conditions had unambiguous values in place of the ambiguous values. For example, Conditions 1–3 were all structurally identical by having a 50%/50% gain/loss choice and a 100% $0 choice, except Condition 1 had no ambiguous values, Condition 2 had ambiguous risky loss, and Condition 3 had ambiguous risky gain; we used the mean of the unambiguous risky gain values from Conditions 1 and 2 to calculate Condition 3’s mean ambiguous risky gain value used in the models, and we used the mean of the unambiguous risky loss values from Conditions 1 and 3 to calculate Condition 2’s mean ambiguous risky loss value used in the models. For Condition 5, we used the mean of the unambiguous risky gain values from Conditions 4 and 6; for Condition 6, we used the mean of the unambiguous sure gain values from Conditions 4 and 5. For Conditions 7 and 8, we used the mean of the values from the unambiguous choice within each of the conditions to calculate the ambiguous means. By assigning free parameter weights to these rational values, we can determine the degree to which participants overestimate or underestimate the value of ambiguous options and thus infer their preference or aversion to ambiguity. The actual values used are shown in ***Table 2***. We chose to use multiplicative weights based on the mean unambiguous values for a few reasons. First, the mean unambiguous values represent rational values/choices. Because participants completed many trials with unambiguous dollar values, they likely learned these unambiguous values and could estimate the values of ambiguous choices. Second, the mean unambiguous values used in our model psychologically represent rational choices, and our multiplicative ambiguity preference/aversion parameters calculate adherence to or deviations from those rational choices. By assigning free parameter weights to these rational mean values, our model parameters allow us to quantify the degree to which participants under- or over-estimate the values of ambiguous options and thus psychologically infer their preference or aversion to ambiguity. For example, if a given participant had an ambiguous risky gain preference parameter value of 1.5 (where 1 indicates treating the ambiguous risky gain rationally), we can conclude that the participant subjectively valued the ambiguous risky gain at 1.5x its rational mean value. To the degree that our experiments and models are externally valid, this 1.5 ambiguous risky gain parameter value suggests that this participant may similarly over-estimate ambiguous risky gains outside of the experiment. Thus, our modeling approach is psychologically grounded by allowing comparison of subjective value and rational value.

**Table 2:** Computational Modeling Calculations.


CONDITION	*U(GAMBLE)* =	*U(SURE)* =

1: Mixed *gain*/loss, unambiguous	0.5 * *gain^ρ^* – 0.5 * λ* (loss)*^ρ^*	0

2: Mixed *gain*/loss, ambiguous risky loss	0.5 * *gain^ρ^*– 0.5 *λ* ***α_RL_*** * (8.15)*^ρ^*	0

3: Mixed *gain*/loss, ambiguous risky *gain*	0.5 * α_RG_* 14.15*^ρ^*– 0.5 * λ* (loss)*^ρ^*	0

4: No–loss, unambiguous	0.5 * *gain^ρ^*	*sure*G*^ρ^*

5: No–loss, ambiguous risky *gain*	0.5 * α_RG_* 15*^ρ^*	*sure*G*^ρ^*

6: No–loss, ambiguous *sure gain*	0.5 * *gain^ρ^*	***α_SG_*** * 5*^ρ^*

7: No risk, ambiguous *sure gain*	***α_SG_*** * 7*^ρ^*	*sureG^ρ^*

8: No risk, ambiguous *sure* loss	–λ* α_SL_* 7*^ρ^*	λ* *sure*L*^ρ^*


Table shows the mean values for ambiguous gains and ambiguous losses for Study 2 (high stakes; $6-$48). Mean values for condition-specific parameters in Study 1 (low stakes; $3.60 to $5.70) are (in cents): Condition 2 (41), Condition 3 (71), Condition 5 (75), Condition 6 (28), Condition 7 (35), and Condition 8 (35).

For each trial, the subjective utilities (*u*) of the gamble and sure option were estimated as follows (with the expected value for each ambiguous condition shown in green for gains and red for losses; see ***Table 2***). λ (“lambda”) represents loss aversion (where λ > 1 indicates overweighting of losses relative to gains, and λ < 1 indicates underweighting losses relative to gains). ρ (“rho”) represents the curvature of the utility function, which reflects exponential changes in sensitivity to values as value increases. If ρ < 1, increases in potential gain values exponentially decreases their subjective utility, indicating risk aversion (i.e., less utility for a gamble than a sure option with the same expected value); if ρ > 1, increases in potential gain values exponentially increases their subjective utility, indicating risk-seeking. α_RG_, α_SG_, α_RL_, and α_SL_ (“alpha risky gain,” “alpha sure gain,” “alpha risky loss,” and “alpha sure loss,” respectively) represent the ambiguity parameters for risky gains, sure gains, risky losses and sure losses, respectively. In the case of gains (α_RG_ and α_SG_), values < 1 mean that ambiguous gain values are underestimated compared to the rational gain, indicating ambiguity aversion, while values > 1 indicate ambiguity preference. In the case of losses (α_RG_ and α_SG_), values > 1 mean that ambiguous loss values are overestimated compared to the rational loss, indicating ambiguity aversion, while values < 1 indicate ambiguity preference. In other words, for parameters with “Aversion” in their name (e.g., Ambiguous Risky Loss Aversion), values > 1 indicate ambiguity aversion, whereas values < 1 indicate preference. Conversely, for parameters with “Preference” in their name (e.g., Ambiguous Risky Gain Preference), values > 1 indicate ambiguity preference, whereas values < 1 indicate ambiguity aversion.

Subjective utility values were then passed through a softmax function to estimate the probability of choosing the gamble on each trial (coded as 1 or 0 for choosing the gamble or the alternative sure option, respectively), with the inverse temperature parameter μ:

\[
P\left({gamble} \right) = \frac{1}{{1 + {e^{ - {\bf{\mu }}\left({u\left[ {gamble} \right]\; - u\left[ {sure} \right]} \right)}}}}
\]

“Gambles” refer to the risky option (Conditions 1–6) or the ambiguous option (Conditions 7–8).

Best-fitting parameters were estimated using a maximum likelihood estimation procedure in MATLAB.

Additional models were determined as follows:

- Model 1: traditional prospect theory model; α_RG_ = α_SG_ = α_RL_ = α_SL_ = 1 (no ambiguity preference or aversion)
- Model 2: single ambiguity parameter; α_RG_ = α_SG_ = α_RL_ = α_SL_ = α
- Model 3: separate ambiguity parameters for gains and losses; α_RG_ = α_SG_ = α_G_ and α_RL_ = α_SL_ = α_L_
- Model 4: separate ambiguity parameters for no-loss contexts (i.e. only values ≥ $0 are present in the trial) and loss context (i.e. at least one loss is present in the trial)
- Model 5: separate ambiguity parameters for ambiguous risky gains (α_RG_), ambiguous sure gains (α_SG_), ambiguous risky losses (α_RL_), and ambiguous sure losses (α_SL_).

### Inferential Statistics

We conducted t-tests to assess differences in gambling percentage/modeling parameters between studies and whether they differed from no preference/aversion. For modeling parameters, a value of “1” indicate no preference/aversion. For Condition 6, we conducted t-tests within Study 1 and 2, where a value of “50” indicates a 50% gambling rate (i.e., no preference/aversion); we then conducted a difference score t-test of Study 2 minus 1, whereas a value of “0” indicates no significant difference in gambling rate between studies. We also conducted multilevel modeling using trait anxiety and depression as Level 2 variables and model parameters as Level 1 variables. We ran separate analyses using just trait anxiety as a predictor of each model parameter and using just trait depression as a predictor of each model parameter. For Bayesian analyses, we used Bayesian linear regression to estimate Bayes factors supporting the null model (i.e., no association between trait anxiety or trait depression and model parameters) and supporting the experimental model (i.e., an association between trait anxiety or trait depression and the model parameters). In these analyses, anxiety or depression were entered in the models separately to assess their effect on model parameters separately. We compared the Bayes factor for the effect of the emotion vs just the intercept (the latter of which is the null model). Bayes factors can be interpreted as the odds that one model (e.g., null model) is supported over the alternative model (e.g., experimental model) (Jarosz & Wiley, 2014; Kass & Raftery, 1995; Quintana & Williams, 2018).

### Data Availability Statement

Please see our GitHub repository for the data and code to replicate our computational modeling, inferential statistics, and figures: *https://github.com/tzbozinek/economic-decision-making-ambiguity*.

## Additional File

The additional file for this article can be found as follows:

10.5334/cpsy.79.s1 Supplemental File. Supplemental materials.
