# Supplementary material for: Economic Decisions with Ambiguous Outcome Magnitudes Vary with Low and High Stakes but Not Trait Anxiety or Depression
Source: Comput Psychiatr. 2021 Oct 21;5(1):119–39. doi: 10.5334/cpsy.79 (PMC11104296; doi:10.5334/cpsy.79)
Supplement: Supplemental File. — Supplemental materials. [file cpsy-5-1-79-s1.pdf]

FULL TITLE: **Economic decisions with ambiguous outcome magnitudes vary with low and high stakes but not trait anxiety or depression**

SUPPLEMENTAL MATERIALS

Tomislav D. Zbozinek* [1], Caroline J. Charpentier* [1], Song Qi [2], and Dean Mobbs[1]

[1]California Institute of Technology, Humanities and Social Sciences, 1200 E. California Blvd., MC 228-77, Pasadena, CA 91125, USA

[2]National Institute of Mental Health, 6001 Executive Boulevard, Room 6200, MSC 9663 Bethesda, MD 20892

*Co-first authors: these authors contributed equally.

Correspondence to:

Tomislav D. Zbozinek, Ph.D.

Division of Humanities and Social Sciences

California Institute of Technology

1200 E. California Blvd., MC 228-77

Pasadena, CA 91125, USA

Email: zbozinek@caltech.edu

METHODS

Participants

Amazon's Mechanical Turk (MTurk)

MTurk is advantageous for being low financial cost and quick for high-quality data collection.(Casler et al., 2013; Hauser et al., 2019) Results from MTurk data replicate those observed in the laboratory across a variety of domains, including judgment and decision making(Paolacci et al., 2010) and economics.(Amir et al., 2012) Amir, Rand, & Gal (2012)(Amir et al., 2012) evaluated the effects of collecting data using MTurk in an economic decision-making study where participants could earn up to $1 bonus payment ($1.40 total); they found their results were comparable to in-person data collection, even with only $1.40 maximum payment. In Study 1, participants started with a base payment of $4.50, and final payment was $3.60-$5.70; in Study 2, participants started with a base payment of $24, and final payment was $6-$48. Based on Amir, Rand, & Gal (2012),(Amir et al., 2012) our payment should be sufficient to observe valid effects in both studies.

Eligibility Criteria

Eligible participants were age 18-65, fluent in English, had healthy or corrected vision, were not colorblind, and had no difficulty controlling motor movements. Because our study used green, red, and white font during the experimental task, we verified whether participants were colorblind using one item from the Ishihara Color Blindness Test (Item 9; shows number "45").(Ishihara, 1960; Melamud et al., 2009) If participants failed this test, their data was excluded from analyses. Additionally, based on relevant suggestions to increase the validity of data collection (for review, see (Hauser et al., 2019)), eligibility criteria also included that participants live in the United States(Litman et al., 2015) and have a ≥95% MTurk Human Intelligence Task (HIT) Approval Ratio.(Peer et al., 2014) Also, to minimize the risk of multiple data entries from the same person, a given MTurk "Worker ID" was only allowed to participate one time in the experiment.

Data Quality Assurance

We enforced several quality assurance checks of the data to maximize its validity. First, we prevented participants from entering responses too quickly on trials in order to facilitate genuine decision making (i.e., responses could only be made ≥1sec after trial onset).(Wood et al., 2017) Second, to exclude inattentive participants, we excluded data for participants who did not respond to ≥10% of trials. Third, participants who pressed "f" or "j" on ≥75% of trials had their data excluded due to automatic responding (counterbalancing of trials results in approximately 50% responding with each key regardless of experimental condition or gambling propensity). Fourth, we excluded participants who failed the colorblindness test. Fifth, we excluded participants with highly invariant choice type (i.e., ≥95% or ≤5% combined no gamble [Conditions 1-6] and unambiguous [Conditions 7-8] choices or combined gambling [Conditions 1-6] and ambiguous [Conditions 7-8] choices). Sixth, participants answered six multiple-choice items assessing their comprehension of the experiment's instructions, English fluency, and attention before completing the gambling task. They needed to answer them 100% correctly, or their data was excluded. Seventh, participants' responses to free-response questions at the end of the experiment (e.g., any problems/feedback regarding the experiment) were evaluated case-by-case, and invalid data was excluded if appropriate (e.g., technical errors).

Materials and Apparatus

Trial Sequence and Counterbalancing

Prior to commencing the 333 experimental trials, there were 33 practice trials – nine in Condition 1; three each in Conditions 2, 3, 5-8; and six in Condition 4 (see trials highlighted in yellow in Fig. SM1). Practice trial sequence was fully randomized, and practice trials were counterbalanced within subjects similar to experimental trials (see below). Practice trials were conducted to familiarize participants with the gambling trials and, because our study focuses on decision making, we included a relatively large number of practice trials with values both at the extremes and near the middle of possible values for each Condition so participants could learn the range of possible gain/loss values before starting the experimental trials.

For the 333 experimental trials, the following factors were counterbalanced within participants: the number of times a) the 50%/50% and 100% gambles (Conditions 1-6) and b) the ambiguous and unambiguous outcome magnitudes (Conditions 7-8) appeared as the left or right choice. Trials were organized into seven blocks to pseudo-randomize trial sequence; Blocks 1-6 had 53 trials each – 13 trials for Condition 1; six each for Conditions 2-4, 7-8; and five each for Conditions 5-6. Block 7 had three trials each for Conditions 1-3, 7-8. Within each block, trial sequence was fully randomized. Between participants, block sequence was fully randomized, and left/right choices were counterbalanced. To dissuade automatic responding, participants were unable to respond <1 sec after trial presentation. Responses were recorded 1-5sec after trial presentation. Trial values were pre-determined with a table of values for each Condition in order to facilitate variability in gambling/not gambling decisions (see Supplemental Materials Figure SM1 below for details). Unambiguous risky gain values ranged from a) 0.64 to 6.67 times the value of unambiguous risky loss values (Condition 1) and b) 0.63 to 3.33 times the value of unambiguous sure gain values (Condition 4). Intertrial intervals were 0.15sec in duration, occurred between every gambling trial, and consisted of a black screen with a white fixation cross in the middle.

Self-Report Questionnaires

See Table SM1 for details on the self-report questionnaires used in this study and Table SM2 for the specific items used in the calculation of the trait anxiety and trait depression composite scores. The following are the equations used to calculate the anxiety and depression composite scores, where the questionnaire name indicates the score for that questionnaire or subscale (average of all items in that questionnaire or subscale):

$$(Eq.\,1) Anxiety\ Composite = \left(\frac{STAI\ Anxiety - 1}{4} * 7 + \frac{DASS21\ Anxiety}{4} * 7 + \frac{PANAS\ Fear - 1}{5} * 6 + \frac{OASIS}{5} * 5\right) * \frac{1}{25}$$

$$(Eq.\,2) Depression\ Composite = \left(\frac{STAI\ Depression - 1}{4} * 13 + \frac{DASS21\ Depression}{4} * 7 + \frac{PANAS\ Sadness - 1}{5} * 5 + \frac{PANAS\ Positive\ Affect, Reverse\ Coded - 1}{5} * 10\right) * \frac{1}{35}$$

Table SM1. Details of the Self-Report Questionnaire Used in the Present Study

Measure	Subscales	Construct	# Items	Reliability (α)	Scale	References
DASS-21	Depression, Anxiety, Stress	Depression, anxiety, physiological stress	21	.81-.97	0-3; 0 = "Does not apply to me," 3 = "Applies to me very much, or most of the time"	Antony, et al., 1998; Lovibond & Lovibond, 1995a, 1995b
OASIS	-	Frequency/intensity of anxiety; impairment due to anxiety	5	.80	0-5; 0 = low/no, 5 = high frequency, severity, or impairment	Norman, et al., 2006
PANAS	General PA, General NA, Fear, Sadness, Hostility	Positive affect, negative affect, fear/anxiety, sadness/depression, hostility/anger	31	.82-.88	1-5; 1 = "Very slightly or not at all," 5 = "Extremely"	Watson & Clark, 1999; Watson, Clark, & Tellegen, 1988
STAI	Total, Anxiety, Depression	Negative affect, anxiety, depression	20	.78-.89	1-4; 1 = "Almost never," 4 = "Almost always"	Bieling, Anton, & Swinson, 1998; Spielberger, 1983

Note: DASS-21 = Depression, Anxiety, and Stress Scale 21; OASIS = Overall Anxiety Severity and Impairment Scale; PANAS = Positive and Negative Affect Schedule; PA = Positive Affect; NA = Negative Affect; STAI = State-Trait Anxiety Inventory.

Table SM2. Questionnaire Items Used in Anxiety and Depression Composite Scores

Anxiety		Depression	
Measure	Item	Measure	Item
DASS-21	I am aware of dryness of my mouth.	DASS-21	I can't seem to experience any positive feeling at all
DASS-21	I experience breathing difficulty (e.g., excessively rapid breathing, breathlessness in the absence of physical exertion).	DASS-21	I find it difficult to work up the initiative to do things
DASS-21	I experience trembling (e.g., in the hands).	DASS-21	I feel that I have nothing to look forward to
DASS-21	I am worried about situations in which I might panic and make a fool of myself.	DASS-21	I feel down-hearted and blue
DASS-21	I feel I am close to panic.	DASS-21	I am unable to become enthusiastic about anything
DASS-21	I am aware of the action of my heart in the absence of physical exertion (e.g., sense of heart rate increase, heart missing a beat).	DASS-21	I feel I am not worth much as a person
DASS-21	I feel scared without any good reason.	DASS-21	I feel that life is meaningless
STAI-T	I feel nervous and restless.	STAI-T	I feel pleasant
STAI-T	I feel that difficulties are piling up so that I cannot overcome them	STAI-T	I feel satisfied with myself
STAI-T	I worry too much over something that really doesn't matter	STAI-T	I wish I could be as happy as others seem to be
STAI-T	I have disturbing thoughts	STAI-T	I feel like a failure
STAI-T	Some unimportant thought runs through my mind and bothers me	STAI-T	I feel rested
STAI-T	I take disappointments so keenly that I can't put them out of my mind	STAI-T	I am "calm, cool, and collected"
STAI-T	I get in a state of tension or turmoil as I think about my recent concerns and interests	STAI-T	I am happy
PANAS Fear	Afraid	STAI-T	I lack self-confidence
PANAS Fear	Shaky	STAI-T	I feel secure
PANAS Fear	Nervous	STAI-T	I make decisions easily
PANAS Fear	Jittery	STAI-T	I feel inadequate
PANAS Fear	Scared	STAI-T	I am content
PANAS Fear	Frightened	STAI-T	I am a steady person
OASIS	How often do you feel anxious?	PANAS Sadness	Sad
OASIS	When you feel anxious, how intense or severe is your anxiety?	PANAS Sadness	Alone
OASIS	How often do you avoid situations, places, objects, or activities because of anxiety or fear?	PANAS Sadness	Blue

OASIS	How much does your anxiety interfere with your ability to do the things you need to do at work, at school, or at home?	PANAS Sadness	Lonely
OASIS	How much has anxiety interfered with your social life and relationships?	PANAS Sadness	Downhearted
		PANAS PA	Attentive
		PANAS PA	Strong
		PANAS PA	Inspired
		PANAS PA	Alert
		PANAS PA	Active
		PANAS PA	Excited
		PANAS PA	Proud
		PANAS PA	Enthusiastic
		PANAS PA	Determined
		PANAS PA	Interested

Note: Questionnaires included are Depression, Anxiety, and Stress Scale - 21-Item Version (DASS-21); State-Trait Anxiety Inventory - Trait Version (STAI-T); Positive and Negative Affect Schedule (PANAS) with its Fear, Sadness, and Positive Affect (PA) Subscales; and Overall Anxiety Severity and Impairment Scale (OASIS). Appropriate items were reverse-coded (e.g., PANAS PA) so that higher values indicated higher anxiety or depression.

Design

Condition Design Matrices

Figure SM1. Condition Design Matrices. Below are the actual US dollar values used in the experiment per Condition in Study 2 (painted in red or green); Study 2 dollar values were 20x of those in Study 1. Thus, the amount that could be gained/lost on a given trial was 20x in Study 2 compared to Study 1. For Conditions 1 and 4, multiplicative values are expressed in each cell (painted in white or yellow) such that the cell values indicate a) the value of the unambiguous risky gain divided by the absolute value of the unambiguous risky loss (Condition 1) and b) the expected value of the unambiguous risky gain divided by the unambiguous sure gain (Condition 4). Higher numbers indicate higher value of the unambiguous risky gain. In both studies, unambiguous risky gain values ranged from a) 0.64 to 6.67 times the value of unambiguous risky loss values (Condition 1) and b) 0.63 to 3.33 times the value of unambiguous sure gain values (Condition 4). Yellow cells were used in both the 333 main trials and the 33 practice trials; white cells were only used in the 333 main trials. Practice trials were selected to demonstrate extreme and central monetary values of each Condition so that learning these values would occur during practice trials and would mitigate learning during main trials; this was done so our main trials would have a relatively greater focus on decision making rather than both learning and decision making.

Condition 1: Risk Aversion, Loss Aversion										
Unambiguous Risky Loss	-$3	**3**	3.5	4	4.5	5	5.5	6.5	7.5	8.5
	-$4	2.5	**3**	3.5	4	4.5	5	6	7	8
	-$5	2	2.5	**3**	3.5	4	4.5	5.5	6.5	7.5
	-$6	1.5	2	2.5	**3**	3.5	4	5	6	7
	-$7	1	1.5	2	2.5	**3**	3.5	4.5	5.5	6.5
	-$8	0.5	1	1.5	2	2.5	**3**	4	5	6
	-$10	-0.5	0	0.5	1	1.5	2	**3**	4	5
	-$12	-1.5	-1	-0.5	0	0.5	1	2	**3**	4
	-$14	-2.5	-2	-1.5	-1	-0.5	0	1	2	**3**
		$9	$10	$11	$12	$13	$14	$16	$18	$20
		Unambiguous Risky Gain								

Condition 2: Risk Aversion, Loss Aversion, Ambiguous Risky Loss Aversion														
Ambiguous Risky Loss	?	?	?	?	?	?	?	?	?	?	?	?	?	?
	?	?	?	?	?	?	?	?	?	?	?	?	?	?
	?	?	?	?	?	?	?	?	?	?	?	?	?	?
		$7	$8	$9	$10	$11	$12	$13	$14	$16	$18	$20	$22	$24
		Unambiguous Risky Gain												

Condition 3: Risk Aversion, Loss Aversion, Ambiguous Risky Gain Aversion														
Ambiguous Risky Gain	?	?	?	?	?	?	?	?	?	?	?	?	?	?
	?	?	?	?	?	?	?	?	?	?	?	?	?	?
	?	?	?	?	?	?	?	?	?	?	?	?	?	?
		-$1	-$2	-$3	-$4	-$5	-$6	-$7	-$8	-$10	-$12	-$14	-$16	-$18
		Unambiguous Risky Loss												

Condition 4: Risk Aversion

Unambiguous Sure Gain						
$3	**2**	3	4	5	6	7
$4	1	**2**	3	4	5	6
$5	0	1	**2**	3	4	5
$6	-1	0	1	**2**	3	4
$7	-2	-1	0	1	**2**	3
$8	-3	-2	-1	0	1	**2**
	$10	$12	$14	$16	$18	$20
	Unambiguous Risky Gain					

Condition 5: Risk Aversion, Ambiguous Risky Gain Aversion

Ambiguous Risky Gain										
?	?	?	?	?	?	?	?	?	?	?
?	?	?	?	?	?	?	?	?	?	?
?	?	?	?	?	?	?	?	?	?	?
	$1	$2	$3	$4	$5	$6	$7	$8	$9	$10
	Unambiguous Sure Gain									

Condition 6: Risk Aversion, Ambiguous Sure Gain Aversion

Ambiguous Sure Gain										
?	?	?	?	?	?	?	?	?	?	?
?	?	?	?	?	?	?	?	?	?	?
?	?	?	?	?	?	?	?	?	?	?
	$6	$8	$10	$12	$14	$16	$18	$20	$22	$24
	Unambiguous Risky Gain									

Condition 7: Ambiguous Sure Gain Aversion														
**Ambiguous Sure Gain**	**?**	?	?	?	?	?	?	?	?	?	?	?	?	?
	**?**	?	?	?	?	?	?	?	?	?	?	?	?	?
	**?**	?	?	?	?	?	?	?	?	?	?	?	?	?
		**$1**	**$2**	**$3**	**$4**	**$5**	**$6**	**$7**	**$8**	**$9**	**$10**	**$11**	**$12**	**$13**
		**Unambiguous Gain**												

Condition 8: Ambiguous Sure Loss Aversion														
**Ambiguous Sure Loss**	**?**	?	?	?	?	?	?	?	?	?	?	?	?	?
	**?**	?	?	?	?	?	?	?	?	?	?	?	?	?
	**?**	?	?	?	?	?	?	?	?	?	?	?	?	?
		**-$1**	**-$2**	**-$3**	**-$4**	**-$5**	**-$6**	**-$7**	**-$8**	**-$9**	**-$10**	**-$11**	**-$12**	**-$13**
		**Unambiguous Sure Loss**												

Procedure

Payment Details

Final monetary compensation was automatically determined by the PsychoPy program. The final trial of the experiment was used to determine participant payment, and, due to randomization of trials and blocks, the order, type, and magnitude of that trial were random. The participants' choice on that trial was used to determine final payment. For trials in which a 100% probability, unambiguous choice was selected, that amount was added to their $4.50 base pay (Study 1) or $24 base pay (Study 2). For trials in which a 50%/50% probability was selected, one of the possibilities was randomly chosen. If an ambiguous value was selected, in Study 1, a random number in $0.05 intervals between $0.05 to $1.20 (ambiguous gain) or -$0.05 to -$0.90 (ambiguous loss) was selected. For Study 2, a random number in $1 intervals between $1 to $24 (ambiguous gain) or -$1 to -$18 (ambiguous loss) was selected. Thus, the amount of money that could be gained or lost on a given decision was 20x in Study 2 vs Study 1. If the participant did not provide a response on that trial, we chose the lowest outcome from that trial using the same methods as above. The range of possible ambiguous values matched the actual range of unambiguous gains/losses. For Study 1, the selected value was added to $4.50 to produce their final payment, ranging from $3.60 to $5.70. For Study 2, the selected value was added to $24 to produce their final payment, ranging from $6 to $48. Importantly, participants were not informed of how the dollar amount of an ambiguous choice would be calculated, nor were they explicitly told the range of possible values for ambiguous choices. This facilitated individual differences in the estimation of the ambiguous dollar values and the comparison of different models and model parameters.

RESULTS

Risk, Loss, and Ambiguity (Model 5) – No Emotions

Tables SM3-5 present details on statistical results that correspond with the confidence interval figures presented in Figure 5 in the main manuscript.

Table SM3. Study 1 ($3.60-$5.70) Emotion-Free Results

Parameter	M	SD	t	p	d	95% CI		Holm-Bonferroni Cutoff
**Loss Aversion**	**2.361**	**2.571**	**10.140**	**<.001**	**.529**	**2.097**	**2.625**	**.008**
Ambiguous Risky Loss Aversion	1.020	.794	.489	.625	.026	.939	1.102	.050
**Ambiguous Sure Loss Aversion**	**1.238**	**.805**	**5.659**	**<.001**	**.295**	**1.155**	**1.321**	**.013**
**Risk Preference**	**1.217**	**.592**	**7.010**	**<.001**	**.366**	**1.156**	**1.278**	**.010**
Ambiguous Risky Gain Preference	1.053	.650	1.550	.122	.081	.986	1.119	.017
**Ambiguous Sure Gain Preference**	**1.842**	**.893**	**18.078**	**<.001**	**.944**	**1.751**	**1.934**	**.007**
Condition 6	51.235	22.841	1.036	.301	.054	48.891	53.580	.025

Positive values indicate greater values of that parameter/condition for Study 1 (low stakes). Significant results in **bold** and gray. Effect size (d) calculated as: d = |(M - "test value")/SD|, where "test value" is 1 for parameters and 50 for Condition 6. Conducted t-tests to compare parameter/gambling value with test value. Holm-Bonferroni cutoff was conducted to correct for multiple analyses.

Table SM4. Study 2 ($6-$48) Emotion-Free Results

Parameter	M	SD	t	p	d	95% CI		Holm-Bonferroni Cutoff
**Loss Aversion**	**2.584**	**2.338**	**9.821**	**<.001**	**.678**	**2.266**	**2.902**	**.008**
Ambiguous Risky Loss Aversion	.952	.639	-1.088	.278	.075	.865	1.039	.025
**Ambiguous Sure Loss Aversion**	**.854**	**.551**	**-3.830**	**<.001**	**.264**	**.779**	**.929**	**.013**
Risk Preference	1.050	.526	1.369	.173	.094	.978	1.121	.017
**Ambiguous Risky Gain Preference**	**.634**	**.371**	**-14.296**	**<.001**	**.986**	**.584**	**.685**	**.007**
Ambiguous Sure Gain Preference	.971	.582	-.722	.471	.050	.892	1.050	.050
**Condition 6**	**63.587**	**25.380**	**7.758**	**< .001**	**.535**	**60.135**	**67.040**	**.010**

Positive values indicate greater values of that parameter/condition for Study 2 (high stakes). Significant results in **bold** and gray. Effect size (d) calculated as: d = |(M - "test value")/SD|, where "test value" is 1 for parameters and 50 for Condition 6. Conducted t-tests to compare parameter/gambling value with test value. Holm-Bonferroni cutoff was conducted to correct for multiple analyses.

Table SM5. Study 2 vs 1 Emotion-Free Results

Parameter	Mean Diff	SD	t	p	d	95% CI		Holm-Bonferroni Cutoff
Loss Aversion	.315	2.628	1.737	.084	.120	-.042	.673	.025
Ambiguous Risky Loss Aversion	-.086	.809	-1.539	.125	.106	-.196	.024	.050
**Ambiguous Sure Loss Aversion**	**-.350**	**.753**	**-6.729**	**<.001**	**.464**	**-.452**	**-.247**	**.013**
**Risk Preference**	**-.183**	**.585**	**-4.545**	**<.001**	**.314**	**-.263**	**-.104**	**.017**
**Ambiguous Risky Gain Preference**	**-.412**	**.649**	**-9.209**	**<.001**	**.635**	**-.500**	**-.324**	**.008**
**Ambiguous Sure Gain Preference**	**-.903**	**.859**	**-15.235**	**<.001**	**1.051**	**-1.020**	**-.786**	**.007**
**Condition 6**	**12.793**	**26.160**	**7.087**	**<.001**	**.489**	**9.235**	**16.352**	**.010**

Conducted t-tests using parameter difference scores of Study 2 minus Study 1 compared to a test value of "0." Positive values indicate greater values of that parameter/condition for Study 2 (high stakes) vs Study 1 (low stakes). Significant results in **bold** and gray. Effect size (d) calculated as: d = |(M - 0)/SD|. Holm-Bonferroni cutoff was conducted to correct for multiple analyses.

Risk, Loss, and Ambiguity (Model 5) – Trait Anxiety and Trait Depression

Tables SM6-9 present details on statistical results that correspond with the confidence interval figures presented in Figure 8 in the main manuscript. There were no significant effects involving trait anxiety or trait depression.

Table SM6. Study 1 ($3.60-$5.70) Anxiety Results

Parameter	b	SE	Z	p	d	95% CI	
Loss Aversion	-.736	.897	-.820	.412	.043	-2.495	1.023
Ambiguous Risky Loss Aversion	-.375	.277	-1.350	.176	.071	-.918	.168
Ambiguous Sure Loss Aversion	-.428	.280	-1.530	.127	.080	-.978	.122
Risk Preference	-.376	.206	-1.820	.068	.095	-.780	.028
Ambiguous Risky Gain Preference	-.186	.227	-.820	.412	.043	-.631	.258
Ambiguous Sure Gain Preference	-.524	.311	-1.690	.092	.088	-1.133	.085
Condition 6	-3.796	7.978	-0.48	.634	.025	-19.433	11.841

Table SM7. Study 2 ($6-$48) Anxiety Results

Parameter	b	SE	Z	p	d	95% CI	
Loss Aversion	-.861	1.093	-.790	.431	.054	-3.004	1.282
Ambiguous Risky Loss Aversion	-.296	.299	-.990	.322	.068	-.881	.289
Ambiguous Sure Loss Aversion	-.417	.257	-1.630	.104	.112	-.920	.085
Risk Preference	-.109	.246	-.440	.659	.030	-.591	.374
Ambiguous Risky Gain Preference	-.071	.174	-.410	.684	.028	-.411	.270
Ambiguous Sure Gain Preference	-.250	.272	-.920	.357	.063	-.784	.283
Condition 6	-1.025	11.889	-0.09	.931	.006	-24.326	22.276

Table SM8. Study 1 ($3.60-$5.70) Depression Results

Parameter	b	SE	Z	p	d	95% CI	
Loss Aversion	.577	.825	0.70	.485	.036	-1.041	2.194
Ambiguous Risky Loss Aversion	.119	.255	0.47	.641	.024	-.381	.619
Ambiguous Sure Loss Aversion	.036	.259	0.14	.890	.007	-.471	.543
Risk Preference	-.121	.190	-0.64	.525	.033	-.493	.252
Ambiguous Risky Gain Preference	-.219	.208	-1.05	.294	.055	-.627	.190
Ambiguous Sure Gain Preference	-.259	.286	-0.90	.366	.047	-.820	.302
Condition 6	-5.690	7.330	-0.78	.438	.041	-20.056	8.675

Table SM9. Study 2 ($6-$48) Depression Results

Parameter	b	SE	Z	p	d	95% CI	
Loss Aversion	.065	.993	0.07	.948	.005	-1.880	2.010
Ambiguous Risky Loss Aversion	-.158	.271	-0.58	.560	.040	-.689	.373
Ambiguous Sure Loss Aversion	2.010	.234	-0.87	.382	.594	-.662	.254
Risk Preference	-.072	.223	-0.32	.747	.022	-.510	.365
Ambiguous Risky Gain Preference	.005	.157	0.03	.977	.002	-.304	.313
Ambiguous Sure Gain Preference	-.099	.247	-0.40	.690	.028	-.583	.385
Condition 6	-.674	10.765	-0.63	.531	.004	-27.842	14.357

Table SM10 shows Bayesian linear regressions estimating the likelihood that the null hypothesis (i.e., no effect of trait anxiety or depression on model parameters) is favored over the experimental hypothesis (i.e., effect of trait anxiety or depression on model parameters). Results show that the data was always in favor of the null hypothesis with moderate odds on average.

Table SM10. Bayesian Linear Regression Results

	Anxiety		Depression	
Parameter	Low Stakes	High Stakes	Low Stakes	High Stakes
Loss Aversion	6.620	4.991	4.150	6.645
Ambiguous Risky Loss Aversion	6.578	4.221	6.057	5.684
Ambiguous Sure Loss Aversion	2.654	1.952	5.722	4.669
Risk Preference	3.132	6.081	6.529	6.343
Ambiguous Risky Gain Preference	6.556	6.164	6.068	6.655
Ambiguous Sure Gain Preference	2.796	4.492	5.155	6.181
Condition 6	6.485	6.635	6.304	5.547
**Parameter Average**	**4.723**	**4.650**	**5.614**	**6.030**

Results show Bayes factor scores for the null model (i.e., no effect of trait anxiety or trait depression), suggesting the ratio of the likelihood that the evidence supports the null hypothesis over the potential effects of trait anxiety or trait depression on model parameters.

Comparing Results Using Study 1's Full Sample vs Using the 210 Participants Who Completed Studies 1 and 2

Below, we assess whether the 157 participants unique to Study 1 influence the results (i.e., whether our results change when using only the 210 participants who were in both the low and high stakes studies). We first re-ran the low stakes model-free analyses with just the N = 210 who were present in both studies. The low stakes results comparing the N = 367 vs N = 210 gambling rates were very similar. We then re-ran the models using the 210 participants who were in both studies, finding that the model fit and parameters results were very similar (Models 3 and 5 shown below). Thus, using the full N = 367 vs the N = 210 participants that are present in both studies does not change the results. Please see Table SM11 below.

Table SM11. Comparison of Results Using Study 1's Full Sample (N = 367) or the Sample That Is Present in Studies 1 and 2 (N = 210)

	N = 367	N = 210	Difference
**Model-Free Gambling Propensity**			
Model-Free Gambling Condition 1	56.36%	57.77%	-1.42%
Model-Free Gambling Condition 2	54.55%	55.23%	-0.67%
Model-Free Gambling Condition 3	50.92%	51.49%	-0.57%
Model-Free Gambling Condition 4	79.81%	79.99%	-0.18%
Model-Free Gambling Condition 5	69.49%	70.00%	-0.51%
Model-Free Gambling Condition 6	51.24%	50.79%	0.44%
Model-Free Gambling Condition 7	70.26%	70.76%	-0.49%
Model-Free Gambling Condition 8	43.08%	44.20%	-1.12%
**Model Fit**			
Model 3 AIC	267.2	264.1	3.090
Model 3 Pseudo-R^2	0.441	.448	-0.007
Model 3 Within-Subjects Accuracy	74.30%	74.65%	-0.35%
Model 3 Between-Subjects Accuracy	63.20%	63.49%	-0.29%
Model 5 AIC	256.4	252.3	4.062
Model 5 Pseudo-R^2	0.473	0.482	-0.009
Model 5 Within-Subjects Accuracy	75.60%	76.08%	-0.48%
Model 5 Between-Subjects Accuracy	65.10%	65.24%	-0.14%
**Model 3 Parameters**			
Loss Aversion	2.388	2.337	0.051
Risk Preference	1.026	1.034	-0.008
Ambiguous Gain Preference	1.388	1.415	-0.027
Ambiguous Loss Aversion	1.087	1.072	0.016
**Model 5 Parameters**			
Loss Aversion	2.361	2.339	0.022
Ambiguous Risky Loss Aversion	1.217	1.219	-0.002
Ambiguous Sure Loss Aversion	1.053	1.047	0.006
Risk Preference	1.020	1.015	0.005
Ambiguous Risky Gain Preference	1.842	1.866	-0.024
Ambiguous Sure Gain Preference	1.238	1.210	0.028

REFERENCES

Amir, O., Rand, D. G., & Gal, Y. K. (2012). Economic Games on the Internet: The Effect of $1 Stakes. *PLoS ONE*, *7*(2), e31461. https://doi.org/10.1371/journal.pone.0031461

Casler, K., Bickel, L., & Hackett, E. (2013). Separate but equal? A comparison of participants and data gathered via Amazon's MTurk, social media, and face-to-face behavioral testing. *Computers in Human Behavior*, *29*(6), 2156–2160. https://doi.org/10.1016/j.chb.2013.05.009

Hauser, D., Paolacci, G., & Chandler, J. (2019). Common concerns with MTurk as a participant pool: Evidence and solutions. In *Handbook of research methods in consumer psychology* (pp. 319–337). Routledge/Taylor & Francis Group. https://doi.org/10.4324/9781351137713-17

Ishihara, S. (1960). *Tests for colour-blindness*. Kanehara Shuppan Company Japan.

Litman, L., Robinson, J., & Rosenzweig, C. (2015). The relationship between motivation, monetary compensation, and data quality among US-and India-based workers on Mechanical Turk. *Behavior Research Methods*, *47*(2), 519–528.

Melamud, A., Hagstrom, S., & Traboulsi, E. (2009). Color vision testing. *Ophthalmic Genetics*, *25*(3), 159–187. https://doi.org/10.1080/13816810490498341

Paolacci, G., Chandler, J., & Ipeirotis, P. G. (2010). *Running Experiments on Amazon Mechanical Turk* (SSRN Scholarly Paper ID 1626226). Social Science Research Network. https://papers.ssrn.com/abstract=1626226

Peer, E., Vosgerau, J., & Acquisti, A. (2014). Reputation as a sufficient condition for data quality on Amazon Mechanical Turk. *Behavior Research Methods*, *46*(4), 1023–1031.

Wood, D., Harms, P. D., Lowman, G. H., & DeSimone, J. A. (2017). Response speed and response consistency as mutually validating indicators of data quality in online samples. *Social Psychological and Personality Science*, *8*(4), 454–464.
